# Practical Chemistry and Pharmacy

**Published:** 1893-12

**Authors:** 

**Affiliations:** LaGrange, Ga.


### PRACTICAL CHEMISTRY AND PHARMACY.

EDITED BY HENRY R. SLACK, M. D., PH. M., LAGRANGE, GA.

### CEMENT FOR CELLULOID.

To mend articles of celluloid, Dr. Steiger recommends moistening the fractured edges with glacial acetic acid, and pressing them firmly together for a few minutes.—*Pharm. Zeit.*

### EXCIPIENT FOR QUININE PILLS.

It is said that lactic acid, in about three drops to fifteen grains of quinine sulphate, makes an excellent excipient for quinine pills. A somewhat larger quantity will be required, if other solid substances are incorporated.—*Drug. Circ.*

### EXCRETION OF MORPHINE BY THE SALIVA.

Rosenthal has ascertained that morphine administered hypodermically, even in daily amounts of no more than 0.05 gramme ($\frac{3}{4}$ gr.), can be clearly and unmistakably detected in the saliva. This secretion, therefore, as being more easily obtainable than the gastric contents, may advantageously be examined instead of the latter in cases of suspected poisoning.—*D. Med. Zeit.*

### TO DETECT ARTIFICIAL COLORING MATTER IN BUTTER.

Shake a small quantity of the butter in alcohol, set aside for two or three minutes, then decant the alcohol and evaporate it over a spirit lamp. There should be no non-volatilizable residue. In case the butter is colored with annetto, a brownish-red sediment forms at the bottom of the dish, which is changed to a blue color by addition of sulphuric acid. If colored with curcumin, a deep rose-colored residue will remain, which is turned brown by treating with hydrochloric acid, and an intense brown by treating with carbonate of potassium or sodium. Saffron produces a sediment that becomes red when treated with lead subacetate, and carrots produce one that turns green with an alkali.—*Merck's Report.*

